# Broccoli Cultivated with Deep Sea Water Mineral Fertilizer Enhances Anti-Cancer and Anti-Inflammatory Effects of AOM/DSS-Induced Colorectal Cancer in C57BL/6N Mice

**DOI:** 10.3390/ijms25031650

**Published:** 2024-01-29

**Authors:** Yeon-Jun Lee, Yanni Pan, Daewoo Lim, Seung-Hwan Park, Sin-Il Sin, KyuBum Kwack, Kun-Young Park

**Affiliations:** 1 Department of Biomedical Science, CHA University, Seongnam 13488, Republic of Korea; iyj1014@naver.com (Y.-J.L.); panyanni@cque.edu.cn (Y.P.); dlaeodn1212@naver.com (D.L.); 2 Agriculture Research Center for Carbon Neutral and Healing, Gurye-gun 57607, Republic of Korea; 3 Graduate School of Integrative Medicine, CHA University, Seongnam 13488, Republic of Korea

**Keywords:** broccoli, cultivation, deep sea water minerals, colon cancer, anticancer

## Abstract

This study aimed to determine the alleviating effect of broccoli grown with deep sea water mineral (DSWM) fertilizer extracted from deep sea water on the development of colorectal cancer in C57BL/6N mice treated with AOM/DSS. Naturaldream Fertilizer Broccoli (NFB) cultured with deep sea water minerals (DSWM) showed a higher antioxidant effect and mineral content. In addition, orally administered NFB, showed a level of recovery in the colon and spleen tissues of mice compared with those in normal mice through hematoxylin and eosin (H&E) staining. Orally administered NFB showed the inhibition of the expression of inflammatory cytokine factors IL-1β, IL-6, TNF, IFN-γ, and IL-12 while increasing the expression of IL-10. Furthermore, the expression of inflammatory cytokines and NF-κB in the liver tissue was inhibited, and that of inflammatory enzymes, such as COX-2 and iNOS, was reduced. In the colon tissue, the expression of p53 and p21 associated with cell cycle arrest increased, and that of Bcl-2 associated with apoptosis decreased. Additionally, the expression of Bax, Bad, Bim, Bak, caspase 9, and caspase 3 increased, indicating enhanced activation of apoptosis-related factors. These results demonstrate that oral administration of broccoli cultivated using DSWM significantly restores spleen and colon tissues and simultaneously inhibits the NF-κB pathway while significantly decreasing cytokine expression. Moreover, by inducing cell cycle arrest and activating cell apoptosis, they also suggest alleviating AOM/DSS-induced colon cancer symptoms in C57BL/6N mice.

## 1. Introduction

Broccoli (*Brassica oleracea* L.) is an annual crop belonging to the cruciferous family (*Brassicaceae*) and is highly nutritious [1]. It is a source of various nutrients, including minerals, vitamins, and dietary fiber, and phytochemicals, including glucosinolates and phenolic compounds. Consequently, a diet rich in broccoli is believed to play a preventive role in chronic conditions, including cardiovascular diseases, carcinogenesis, breast cancer, and prostate cancer [2,3,4]. Furthermore, broccoli has an antioxidant activity that helps prevent oxidative stress associated with several diseases [5]. Particularly, because of the effects of broccoli, plant-based bioactive compounds, such as glucosinolates and their breakdown products, have received increasing attention [2,6]. These plant-derived nutrients are essential for human nutrition and health, and the mineral content and phytochemical composition of plants are influenced by various factors, including cultivation methods, fertilizers, and environmental conditions [7,8]. Particularly, plants need nutrients, including essential nutrients (N, P, K, Ca, Mg, Mn, Fe, B, Zn, etc.) and beneficial nutrients (Se, Si, Na, etc.) for growth and development [9]. If either one is lacking, plant growth and production will decrease. Additionally, soil conditions affect plant growth. Particularly, if it is affected by salt or lacks nutrients, it may cause nutrient deficiency or some physiological disorders in the plant [10]. Thus, soil and nutrients significantly affect plant growth, and existing plant cultivation methods have raised concerns about sustainability and environmental pollution due to the lack of nutrients in the soil and the continuous use of chemical fertilizers [11]. Therefore, the iCOOP Natural Dream Co. (Goesan, Chungcheongbuk-do, Republic of Korea) has developed a cultivation method based on organic farming that uses natural minerals extracted from deep-ocean water as fertilizer to fill in insufficient nutrients in the soil. Previous studies have confirmed the in vitro anti-cancer and anti-inflammatory effects of broccoli grown using deep sea water minerals [12].

Colorectal cancer is a very common malignant tumor observed globally [13]. Colorectal cancer affects more than one million people yearly, and despite improvements in early screening and treatment, it remains the fourth leading cause of tumor-related death [14]. Colon cancer is mostly caused by environmental risk factors rather than genetic changes, such as chronic intestinal inflammation, mutagenic substances in food products, and intestinal pathobiont [15]. Among these environmental factors, chronic inflammation is the most important risk factor for colon cancer. Inflammatory responses play a well-known role in tumor development, and long-term inflammatory bowel diseases, including Crohn’s disease and ulcerative colitis, which are associated with colorectal cancer, are also associated with a 1.4- to 2.2-fold increased risk of colorectal carcinoma [16]. Most colonic tumors arise from the epithelium, undergoing a process known as epithelial–mesenchymal transition (EMT) in which they lose their epithelial phenotype and simultaneously acquire a mesenchymal phenotype, providing them with a metastatic capacity [17]. EMT performs a crucial function in cancer development because it leads to the destruction of intercellular adhesions, extracellular matrix remodeling, and increased motility of tumor cells, and tumorigenesis and metastasis are the most influential in the development of colorectal cancer [18]. Particularly, tumor-related inflammation shows no sign in the early stages of colon cancer; therefore, it is often diagnosed at an advanced stage, making patient survival and treatment difficult [19,20]. Therefore, an AOM/DSS-induced colorectal-cancer-related mouse model was created to determine the pathogenesis, drug discovery, and validation of new therapeutic targets for colorectal cancer [21]. Additionally, based on this model, the importance of colon cancer and inflammatory processes was verified, and the mechanism of colon cancer development related to intestinal inflammation was revealed, focusing on the functions of proinflammatory and anti-inflammatory cytokines [22,23]. Particularly, the possible genetic mechanisms of inflammation-induced colorectal cancer include adenomatous polyposis coli, loss of function of tumor suppressor genes such as p53, and increased expression of inflammatory genes such as COX-2 and NOS [24]. Inflammation also involves interactions between various proinflammatory mediators that can trigger signaling for tumor cell proliferation, growth, and invasion [25]. Therefore, this study aimed to determine whether broccoli grown with deep sea water mineral fertilizer suppresses inflammation and colon cancer in mice with AOM/DSS-induced colon cancer.

## 2. Results

### 2.1. Antioxidant Capacities and Mineral Analysis of Broccoli Samples

**Figure 1:**
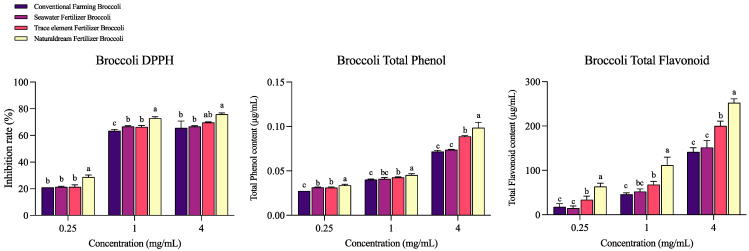
DPPH free radical scavenging, total phenol, and total flavonoid contents by the concentration of broccoli. Means with different letters (a–c) above the bars are significantly different (*p* < 0.05) using Duncan’s multiple range test.

The antioxidant activity of broccoli was evaluated by determining the DPPH free radical scavenging rate and the total polyphenol and flavonoid contents of broccoli cultivated with fertilizers with different mineral contents. The experimental results (Figure 1) revealed that the DPPH free radical scavenging ability of broccoli cultivated with Naturaldream fertilizer (NFB) was the highest at all concentrations, followed by broccoli cultivated with trace element fertilizer (TFB). For the TF content, the NFB group was the highest, followed by the TFB group. At all concentrations, the TP content also showed the highest trend in the NFB group, which differed significantly from the TFB, SFB, and CFB groups (*p* < 0.05). In addition, because of mineral analysis among broccoli (Supplemental Table S2), NFB showed higher magnesium, calcium, and potassium contents than CFB, SFB, and TFB groups, and iron and copper, which are the trace elements, also showed the highest contents (*p* < 0.05).

### 2.2. Mouse Body and Organ Weight, Colon Length, Number of Tumors, and Colon Weight/Length Ratio

**Figure 2:**
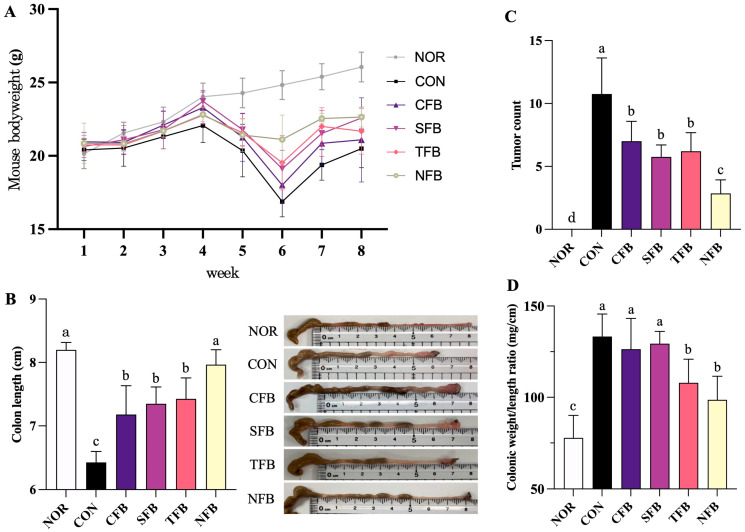
Effect of broccoli oral administration on body weight, colon length, tumor count, and colon weight/length ratio in C57BL/6 mice with colon cancer induced by AOM/DSS. NOR: saline solution; CON: AOM/DSS + saline solution; CFB: AOM/DSS + Conventional Farming Broccoli 1.4 g/kg b.w.; SFB: AOM/DSS + Seawater Fertilizer Broccoli 1.4 g/kg b.w.; TFB: AOM/DSS + Trace element Fertilizer Broccoli 1.4 g/kg b.w.; NFB: AOM/DSS + Naturaldream Fertilizer Broccoli 1.4 g/kg b.w. (**A**) Mice body weight, (**B**) Colon length of the mice, (**C**) Tumor count of the mice, and (**D**) Mice colonic weight/length ratio. Means with different letters (a–d) above the bars are significantly different (*p* < 0.05) using Duncan’s multiple range test.

Figure 2A shows the changes in the body weight of the mice. At the onset of the experiment, the average weight of all mice was 20.6 ± 0.3 g, and all the groups were indifferent. During the experiment, the weight of the NOR group increased continuously. In the AOM/DSS treatment group, the weight gradually decreased after the 1-week period of 2% DSS drinking water treatment on the third week, and the lowest weight was reached on the sixth week. However, broccoli samples alleviated the weight loss phenomenon in mice to varying degrees, with NFB having the best effect, followed by TFB. The weights of the liver, kidneys, and testes were measured to ensure that acute ulcerative colorectal cancer was accurately targeted, and the spleen’s size was compared according to the body’s inflammatory response. For the liver, kidney, and testis, it was confirmed that there were insignificant differences between the NOR and AOM/DSS treatment groups. For the spleen, the CON group treated with AOM/DSS showed the highest weight, and the weight was reduced in the order of the CFB, SFB, TFB, and NFB groups orally administered with broccoli samples (Table 1). The colon length of the CON group treated with AOM/DSS was 6.4 ± 0.1 cm, which was significantly decreased compared with that of the NOR group (8.2 ± 0.1 cm), confirming cancer induction (*p* < 0.05). However, in the groups treated with AOM/DSS and those orally administered broccoli for eight weeks, the length of the large intestine increased significantly, with the NFB group showing the highest similarity to the NOR group at 7.9 ± 0.2 cm. The colon length of mice in the TFB, SFB, and CFB groups were shortened sequentially without a significant difference (Figure 2B). Tumors in the mouse colon tissue were absent in the NOR group, 10.8 ± 2.9 in the CON group, 7.0 ± 1.6 in the CFB group, 5.8 ± 1.0 in the SFB group, 6.2 ± 1.5 in the TFB group, and 2.7 ± 1.1 in the NFB group. Among the groups treated with AOM/DSS, the NFB group showed the highest reduction (Figure 2C). Figure 2D shows that AOM/DSS increased the colon weight/length ratio of mice compared with the NOR group, whereas broccoli intake reduced the colon weight/length ratio of mice, with the NFB group of 98.6 ± 12.9 mg/cm being the lowest among the AOM/DSS treatment groups. These results indicate that broccoli has delaying and inhibiting effects on AOM/DSS-induced colorectal cancer and can effectively inhibit tumor growth and colon contraction during colorectal cancer development. Furthermore, it shows that it can effectively alleviate the immune response by decreasing the spleen’s weight.

### 2.3. Morphological Changes in the Mice Colon and Spleen Tissues

**Figure 3:**
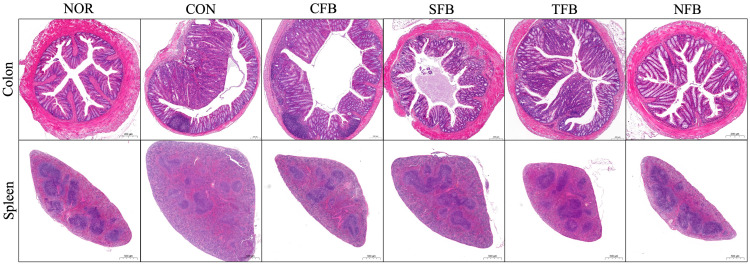
Morphological changes in mice colon and spleen tissues after administration of broccoli samples. NOR: saline solution; CON: AOM/DSS + saline solution; CFB: AOM/DSS + Conventional Farming Broccoli 1.4 g/kg b.w.; SFB: AOM/DSS + Seawater Fertilizer Broccoli 1.4 g/kg b.w.; TFB: AOM/DSS + Trace element Fertilizer Broccoli 1.4 g/kg b.w.; NFB: AOM/DSS + Naturaldream Fertilizer Broccoli 1.4 g/kg b.w.

The pathological results of the mouse colon showed that the normal group was not structurally damaged, and the villi were arranged neatly and regularly, whereas the mucosa of the AOM/DSS group had tumors and inflammatory infiltration, and the villi were disorderly arranged. The villi in the CFB group tended to recover neatly but showed similar inflammatory infiltration to that in the CON group. The visible inflammatory sites in the SFB and TFB groups were reduced, and the colon tissue of the mice in the NFB group recovered to the extent most similar to that in the normal group (Figure 3). Pathological changes in the spleen were observed through the white and red pulps. The normal group spleen section displayed normal splenic architecture, including normal periarterial lymphatic sheaths, lymphoid follicles, and sinuses. The control group showed reduced lymphoid follicles and distorted lymphoid architecture with diffuse white pulp. Broccoli-treated groups demonstrated relatively normal splenic structure, with the CFB and SFB groups showing relatively normal spleen size and clear boundaries of white and red pulps compared with the CON group, while the NFB group showed the clearest white and red pulps, which was most similar to the normal group (Figure 3).

### 2.4. Levels of Inflammation-Related Cytokines Secreted by Mice Blood Serum

**Figure 4:**
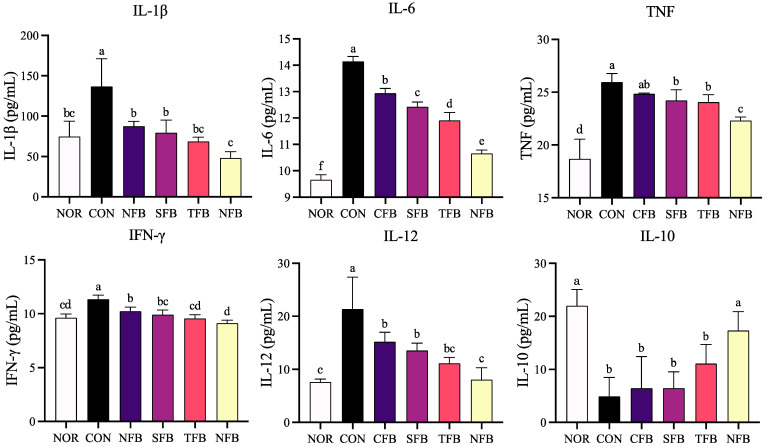
Concentration of the cytokines IL-1β, IL-6, TNF, IFN-γ, IL-12, and IL-10 in mice blood serum. NOR: saline solution; CON: AOM/DSS + saline solution; CFB: AOM/DSS + Conventional Farming Broccoli 1.4 g/kg b.w.; SFB: AOM/DSS + Seawater Fertilizer Broccoli 1.4 g/kg b.w.; TFB: AOM/DSS + Trace element Fertilizer Broccoli 1.4 g/kg b.w.; NFB: AOM/DSS + Naturaldream Fertilizer Broccoli 1.4 g/kg b.w. Means with different letters (a–f) above the bars are significantly different (*p* < 0.05) using Duncan’s multiple range test.

When colon cancer was induced with AOM/DSS, the expression of inflammation-related genes such as IL-1β, IL-6, and TNF in the blood increased, and the expression of inflammatory cytokines was confirmed in the serum of mice. IL-1β, IL-6, TNF, IFN-γ, and IL-12, which affect immunity and inflammation and are related to inflammation, all showed the highest levels of inflammation in the control group, and the groups that were orally given broccoli exhibited a significant decreasing trend. Among them, the expression levels of IL-1β, IFN-γ, IL-6, and TNF in the NFB group were most similar to those in the NOR group, and those of IL-12 in the SFB, TFB, and NFB groups were low. In terms of IL-10 expression related to anti-inflammatory activity, it was verified that the expression level increased in the ionic mineral group to a level that was most similar to that in the normal group (Figure 4).

### 2.5. Levels of Inflammation-Related Cytokines Secreted by Mice Splenocytes

**Figure 5:**
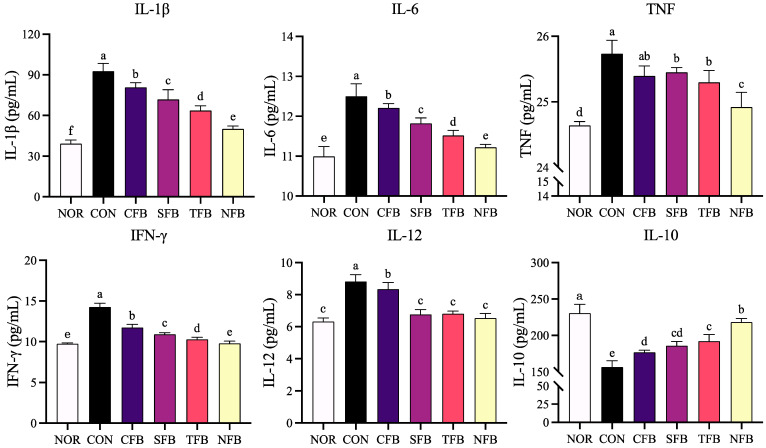
Concentration of the cytokines IL-1β, IL-6, TNF, IFN-γ, IL-12, and IL-10 in mice splenocytes. NOR: saline solution; CON: AOM/DSS + saline solution; CFB: AOM/DSS + Conventional Farming Broccoli 1.4 g/kg b.w.; SFB: AOM/DSS + Seawater Fertilizer Broccoli 1.4 g/kg b.w.; TFB: AOM/DSS + Trace element Fertilizer Broccoli 1.4 g/kg b.w.; NFB: AOM/DSS + Naturaldream Fertilizer Broccoli 1.4 g/kg b.w. Means with different letters (a–f) above the bars are significantly different (*p* < 0.05) using Duncan’s multiple range test.

The levels of inflammation-related cytokines IL-1β, IL-6, TNF, IFN-γ, and IL-12 were the highest in the splenocyte culture supernatant of mice in the control group treated with AOM/DSS. In the broccoli treatment group, a significant reduction trend was observed, and the levels of the above-mentioned inflammatory cytokines in the NFB group decreased similarly to that in the NOR group. Regarding the expression of IL-10 related to anti-inflammation, the broccoli treatment group showed an upward trend. The expression level of the NFB group was similar to that of the normal group (Figure 5).

### 2.6. Effect of Broccoli on NK Cell Activity in Mice Splenocytes

**Figure 6:**
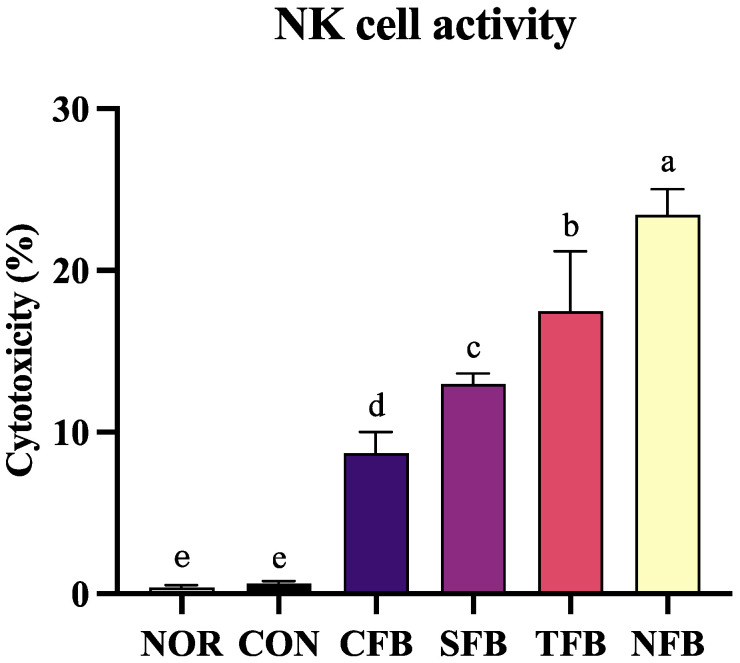
NK cell activity in mice splenocytes. NOR: saline solution; CON: AOM/DSS + saline solution; CFB: AOM/DSS + Conventional Farming Broccoli 1.4 g/kg b.w.; SFB: AOM/DSS + Seawater Fertilizer Broccoli 1.4 g/kg b.w.; TFB: AOM/DSS + Trace element Fertilizer Broccoli 1.4 g/kg b.w.; NFB: AOM/DSS + Naturaldream Fertilizer Broccoli 1.4 g/kg b.w. Means with different letters (a–e) above the bars are significantly different (*p* < 0.05) using Duncan’s multiple range test.

The activity of NK cells in the splenocytes of mice in the normal and control groups was almost not reflected, but that of NK cells in the mice orally administered broccoli was reflected to a certain extent. In the following order in the CFB, SFB, TFB, and NFB groups, the activity of NK cells in splenocytes increased gradually (*p* < 0.05), and that in the NFB group was higher than 20% (Figure 6).

### 2.7. Effects of the Samples on the mRNA and Protein Expression of Inflammation-Related Genes in Mice Liver Tissue

**Figure 7:**
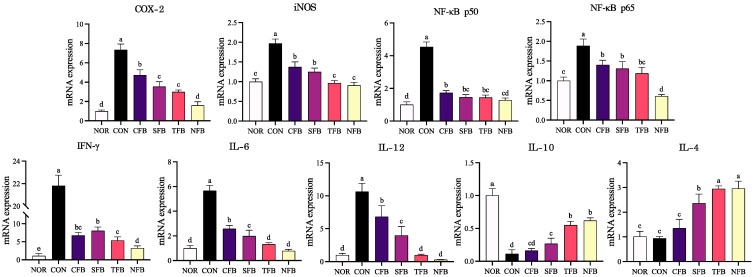
mRNA expression levels of inflammation-related genes in mice liver tissue. NOR: saline solution; CON: AOM/DSS + saline solution; CFB: AOM/DSS + Conventional Farming Broccoli 1.4 g/kg b.w.; SFB: AOM/DSS + Seawater Fertilizer Broccoli 1.4 g/kg b.w.; TFB: AOM/DSS + Trace element Fertilizer Broccoli 1.4 g/kg b.w.; NFB: AOM/DSS + Naturaldream Fertilizer Broccoli 1.4 g/kg b.w. Means with different letters (a–e) above the bars are significantly different (*p* < 0.05) using Duncan’s multiple range test.

**Figure 8:**
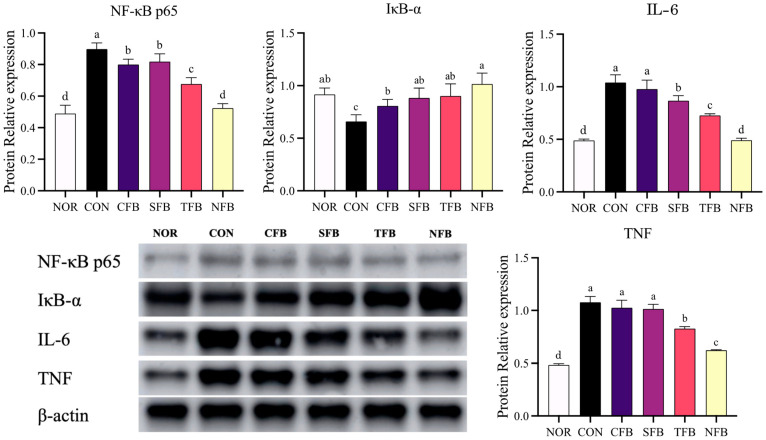
Protein analysis of inflammation-related genes in the mice liver tissue. NOR: saline solution; CON: AOM/DSS + saline solution; CFB: AOM/DSS + Conventional Farming Broccoli 1.4 g/kg b.w.; SFB: AOM/DiSS + Seawater Fertilizer Broccoli 1.4 g/kg b.w.; TFB: AOM/DSS + Trace element Fertilizer Broccoli 1.4 g/kg b.w.; NFB: AOM/DSS + Naturaldream Fertilizer Broccoli 1.4 g/kg b.w. Means with different letters (a–d) above the bars are significantly different (*p* < 0.05) using Duncan’s multiple range test.

RT-qPCR and Western blotting were performed to measure the expression of inflammation-related mRNA and protein in the liver tissue of AOM/DSS-induced colorectal cancer (CRC) mice. The mRNA expression levels of inflammatory markers, including NF-κB, IFN-γ, COX-2, iNOS, IL-6, and IL-12, were significantly upregulated in AOM/DSS-treated mice (Figure 7) (*p* < 0.05). Conversely, it showed a gradual decrease in AOM/DSS-treated mice that consumed broccoli extract orally, and in particular, mice that consumed NFB showed a low expression level similar to that of the NOR group (*p* < 0.05). The anti-inflammatory factors IL-10 and IL-4 were decreased in the AOM/DSS treatment group compared with the NOR group, and the group administered orally with TFB and NFB demonstrated recovery similar to the NOR group (*p* < 0.05). Changes in protein expression of inflammatory markers, including NF-κB p65, IL-6, and TNF, were significantly increased in AOM/DSS-induced CRC mice (Figure 8) (*p* < 0.05). This expression tended to decrease with broccoli extract consumption, with NFB showing the lowest expression; this result exhibited a similar trend to mRNA expression. Thus, it appears that the oral administration of broccoli using DSWM as fertilizer decreases the mRNA and protein expression of inflammatory factors in the liver compared with the others, showing a higher anti-inflammatory effect than other samples.

### 2.8. Effects of the Samples on the mRNA and Protein Expression of Apoptosis-Related Genes in Mice Colon Tissue

**Figure 9:**
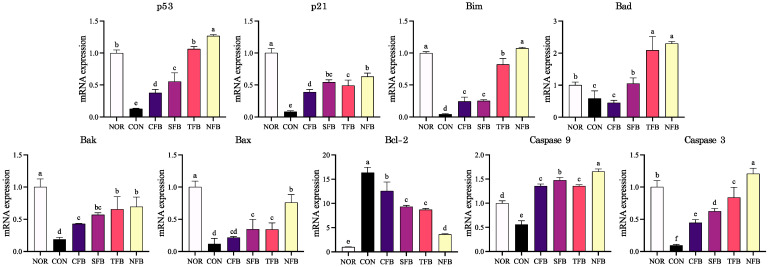
mRNA expression levels of apoptosis-related genes in mice colon tissue. NOR: saline solution; CON: AOM/DSS + saline solution; CFB: AOM/DSS + Conventional Farming Broccoli 1.4 g/kg b.w.; SFB: AOM/DSS + Seawater Fertilizer Broccoli 1.4 g/kg b.w.; TFB: AOM/DSS + Trace element Fertilizer Broccoli 1.4 g/kg b.w.; NFB: AOM/DSS + Naturaldream Fertilizer Broccoli 1.4 g/kg b.w. Means with different letters (a–f) above the bars are significantly different (*p* < 0.05) using Duncan’s multiple range test.

**Figure 10:**
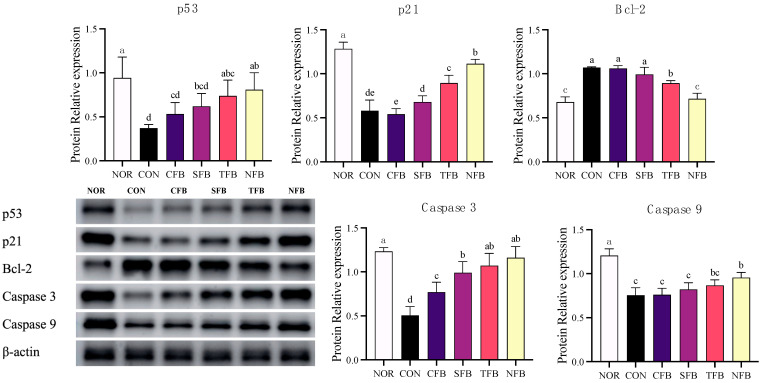
Protein analysis of apoptosis-related genes in the mice colon tissue. NOR: saline solution; CON: AOM/DSS + saline solution; CFB: AOM/DSS + Conventional Farming Broccoli 1.4 g/kg b.w.; SFB: AOM/DSS + Seawater Fertilizer Broccoli 1.4 g/kg b.w.; TFB: AOM/DSS + Trace element Fertilizer Broccoli 1.4 g/kg b.w.; NFB: AOM/DSS + Naturaldream Fertilizer Broccoli 1.4 g/kg b.w. Means with different letters (a–e) above the bars are significantly different (*p* < 0.05) using Duncan’s multiple range test.

To elucidate the mechanism of tumor cell death, the mRNA expression of cell cycle arrest and apoptosis-related genes in the colon of AOM/DSS-treated C57BL/6 mice was measured using RT-qPCR (Figure 9). AOM/DSS treatment reduced the expression of cell cycle arrest-related genes, including p21 and p53, and apoptosis-related factors, including Bim, Bad, Bak, Bax, caspase-9, and caspase-3, in the colon tissue of mice. There was a significant upregulation trend in the mouse groups that were orally administered broccoli extract, with the NFB group showing recovery similar to the NOR group. The expression of Bcl-2, which is related to anti-apoptosis, increased when treated with AOM/DSS and was significantly downregulated in the groups treated with broccoli extract (*p* < 0.05). For more detailed results, the protein expression of apoptosis-related factors was measured using Western blotting (Figure 10). Similar to mRNA expression, AOM/DSS treatment reduced the expression of p53, p21, caspase-3, and caspase-9 and increased the expression of Bcl-2. Additionally, the protein expression of p53, p21, caspase-3, and caspase-9 increased significantly in the group administered broccoli extract orally, with p21 and caspase-9 being the most upregulated in NFB (*p* < 0.05). These results suggest that the oral administration of broccoli extract in mice with AOM/DSS-induced CRC promotes CRC suppression by modulating cell cycle arrest and apoptosis-related factors, particularly demonstrating higher inhibitory effects in broccoli cultivated with DSWM.

### 2.9. Metabolomics Analysis and PLS-DA Score Plot

**Figure 11:**
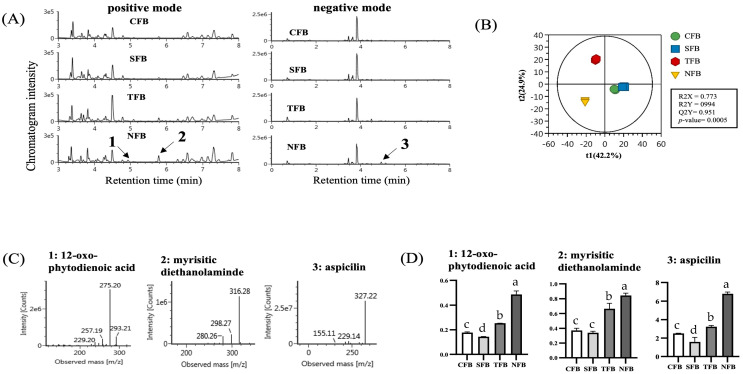
Metabolomic analysis of broccoli and relative abundances of major compounds. Metabolomic profiles of broccoli were analyzed using ultra-performance liquid chromatography-quadrupole time-of-flight mass spectrometry (UPLC-Q-TOF MS) with positive and negative modes (**A**) and a PLS-DA score plot was used to visualize the difference between samples (**B**). The major metabolites that contributed to the difference on the score plot were tentatively identified by the UNIFI software connected to various online databases (**C**) and their relative abundances were compared (**D**). Means with different letters (a–d) above the bars are significantly different (*p* < 0.05) using Duncan’s multiple range test.

Metabolite profiling between broccoli samples was performed using UPLC-Q-TOF MS, and differences between samples were visualized via PLS-DA. PLS-DA score plots of positive and negative LC-MS data clearly demonstrate sharp separation between all sample groups with statistically acceptable quality parameters (R2X = 0.773, R2Y = 0994, Q2Y = 0.951, *p*-value = 0.0005). Additionally, the cross-validation values determined by permutation tests suggested the statistical validity of the PLS-DA model. The results showed significant separation between broccoli and indicated that changes in metabolite profiles contributed to the distinct separation in the PLS-DA score plots (Figure 11B). Additionally, the major metabolites that contributed to the differences in the changed score plots in positive and negative modes were tentatively identified by UNIFI version 1.9.2.045 software linked to various online databases (Figure 11C) and their relative abundances were compared (Figure 11D). As a result of the analysis, substances such as 12-oxo-phytodienoic acid, myrisitic diethanolamine, and aspicilin were analyzed, and the highest content was found in NFB grown with DSWM fertilizer (*p* < 0.05).

## 3. Discussion

This study aimed to investigate the anti-inflammatory and anticancer effects of broccoli grown using deep sea water minerals (NFB) on the development of AOM/DSS-induced colon cancer. Recently, it was confirmed that NFB inhibited the growth of HT-29 human colon cancer cells by regulating cell cycle inhibition and apoptosis-related pathways and significantly regulated the expression of inflammatory cytokines in C57BL/6N mouse splenocytes [12]. Over the past two decades, several chemically induced CRC models, such as mouse models of colorectal carcinogenesis, have been developed. The AOM/DSS-induced mouse model is a very commonly used animal model for the recapitulation of CRC pathogenesis in patients [24,26]. Therefore, an AOM/DSS-induced colon cancer model was constructed in C57BL/6 mice.

Mice with AOM/DSS-induced CRC exhibited decreased body weight gain, bloody stools, shortened colon length, and morphological changes. Ultimately, this increased the colon weight and tumor number, with a higher weight-to-length ratio of the colon [26,27,28]. Similar to these findings, in this study, body weight was reduced in AOM/DSS-induced CRC mice, and colon length was shortened. The number of tumors increased, and the colon weight/length ratio tended to increase compared with that in normal mice. According to existing epidemiological studies, broccoli reduces the risk of cancer, and sulforaphane, the main component of broccoli, has been verified to possess anti-inflammatory and anticancer features [29]. Sulforaphane significantly inhibited the expression of inflammatory cytokines in colon carcinomas and alleviated ulcerative colitis induced by DSS in mice [30,31]. Owing to the oral administration of broccoli extract to mice with AOM/DSS-induced CRC, it was confirmed that the colon lengthened, the colon weight/length ratio reduced, and the number of tumors reduced. DSS dissolved in drinking water is toxic to the epithelial lining of the large intestine, causing severe colitis, which is characterized by weight loss and bloody stool after DSS ingestion, and causes inflammation in the liver and spleen, causing weight gain [32,33]. Accordingly, in this study, the weight of the liver and spleen of the CON group increased significantly compared with that of the NOR group and was decreased in the groups that consumed broccoli extract. Particularly, alleviation was most pronounced in broccoli cultivated with DSWM fertilizer in the NFB group.

Histopathology is a common method for assessing intestinal inflammation and pathology in animal models, typically revealing moderate to severe dysplasia in the epithelium of mouse tissues treated with AOM/DSS. This is accompanied by irregular crypt arrangement, clear infiltration of inflammatory cells in the mucosa, and the formation of typical tumors in the distal colon [33]. In this histopathological analysis, epithelial cell loss, crypt loss, and adenocarcinoma occurred in the control group; however, these symptoms were alleviated in the broccoli-treated group, especially the NFB group, showing similar features to the normal group. Splenomegaly was associated with a reduction in white pulp in tumor-bearing animals [34]. Compared with the spleen tissue of the NOR group, where the white and red medulla were clearly distinguished, AOM/DSS-treated mice exhibited spleen enlargement and the disappearance of the border between the red and white pulps. After broccoli treatment, there was a significant improvement in the shape of the spleen tissue. Particularly, the NFB group demonstrated a shape similar to that of the normal group, suggesting that NFB improves abnormal tissue morphology. 

While inflammation mediates protective responses against pathogen infection and tissue damage, chronic inflammation causes problems associated with oncogenic events, particularly tumor formation in the gastrointestinal tract [35]. Chronic intestinal inflammation represents an early stage in CRC development, and numerous studies have shown that patients with inflammatory bowel disease (IBD) have an approximately 2-to-3-fold increased risk of CRC compared with healthy individuals [36]. Inflammation also causes gut dysbiosis, which can contribute to various pathological conditions, including IBD and CRC, by producing reactive metabolites and carcinogens and disrupting the epithelial barrier [37]. These effects may upset the balance between proinflammatory mediators (e.g., tumor necrosis factor [TNF], interleukin [IL]-1, and IL-6) and anti-inflammatory signals (e.g., IL-10, transforming growth factor (TGF)-ß) in enterocytes and cells of the intestinal immune system [38]. These inflammatory cytokines often act through the activation of the transcription factors nuclear factor κ light-chain enhancer (NF-κB) and STAT3 [39,40]. It was demonstrated that the activation of NF-κB by the IκB kinase (IKK) complex contributes to tumor initiation and growth by blocking enterocyte apoptosis and inducing the production of proinflammatory mediators in macrophages [41]. NF-κB, an inducible transcription factor, can induce the expression of inflammatory cytokine genes (e.g., TNF, IL-1β, IL-6, and IL-8) and inflammatory enzymes (e.g., COX-2 and iNOS) and regulate cell proliferation (e.g., cyclin D1 and c-Myc), survival, and differentiation [42,43]. Nitric oxide (NO), an inflammatory mediator, plays an essential role in physiological processes such as neurotransmission, vasodilation, and immune defense. However, excessive NO production owing to increased iNOS expression is a characteristic of chronic inflammatory diseases of the gastrointestinal tract [44,45]. COX-2, an inflammatory enzyme, is essential in regulating inflammatory responses and lipid metabolism, and given its functional role, it may be associated with inflammation and lipid metabolism and contribute to colon tumorigenesis [27]. 

Therefore, blocking these cytokines prevents CRC progression. In particular, IL-1β plays a central role in mediating inflammatory responses, and it was found that blocking IL-1β activity significantly reduced mucosal damage and tumor formation in a colitis-associated CRC (CAC) mouse model [46]. Furthermore, as part of the interactions among inflammatory cytokines, TNF, as such a mediator, can induce IL-6 expression in colorectal carcinomas, inducing therapeutic benefits in CRC clinical trials through IL-6 inhibition and anti-TNF therapies [47,48,49]. IL-12 is a heterodimeric cytokine produced primarily by macrophages and dendritic cells and stimulates the production of IFN-γ and TNF in T cells and decreases IL-4-mediated inhibition [50]. Additionally, IL-10 is an anti-inflammatory cytokine that can suppress several inflammatory responses and is an important factor in maintaining immune response homeostasis in many human diseases [51].

According to the findings of this study, broccoli extract inhibited the expression of NF-κB, COX-2, iNOS, IL-1β, IL-6, IL-12, TNF, and IFN-γ while increasing the expression of IL-10 and IL-4. These results showed that broccoli extract was effective in restoring inflammatory balance in a CRC mouse model, which appears to be due to the anticancer and anti-inflammatory effects of broccoli as well as the influence of sulforaphane, the main component of broccoli. In particular, sulforaphane regulates the expression of cytokines in colon carcinoma cells and has a significant effect on the prevention and development of cancer on the interaction between immune cells and colon cancer cells [31]. Among these broccoli extracts, it was confirmed that the NFB group tended to have the lowest expression. When broccoli is cultivated using mineral fertilizer, it significantly influences high yield, quality, nutrient content, and sulforaphane levels. Through these results, it is implied that broccoli cultivated with DSWM fertilizer can significantly inhibit inflammatory responses [52,53,54].

Numerous research data strongly support the hypothesis that daily broccoli consumption contributes to reduced cancer risk [55,56,57]. Cancer progression is a multifactorial process involving cellular mutations, leading to unimpeded cell proliferation, subsequently resulting in detrimental effects on the body through malignant cell invasion and distant metastasis, culminating in widespread organ dysfunction [58]. The inhibition of carcinoma proliferation is a critical component of the anticancer mechanism, and regulating the cell cycle and promoting apoptosis are key strategies to effectively inhibit carcinomas [59,60]. When DNA damage or other stressful conditions occur, p53 is activated and triggers a series of responses, including cell cycle arrest, DNA repair, and apoptosis induction [61]. As a downstream target gene of p53, p21 is a cell cycle regulator protein that can inhibit cell cycle progression, preventing cell proliferation and DNA replication, thereby guiding cells toward apoptosis [62]. 

The Bcl-2 family of proteins, closely associated with apoptosis, can be classified into three main categories: the anti-apoptotic protein subfamily, which includes Bcl-2, Bcl-xL, Mcl-1, etc.; the pro-apoptotic protein subfamily, which includes Bax, Bak, Bok, etc.; and another subclass consisting of pro-apoptotic proteins containing only the BH3 domain, known as the BH3-only subfamily, which includes Bid, Bim, Bad, etc. The BH3-only subfamily proteins, Bim and Bad, play important roles in regulating apoptosis. [63,64,65,66,67]. They bind to Bcl-2, inhibiting its anti-apoptotic function [68]. This results in the release or activation of pro-apoptotic proteins, such as Bax and Bak, which directly interact with the outer mitochondrial membrane, increasing its permeability [69]. This leads to the release of apoptotic factors, such as cytochrome C, from the mitochondria, ultimately triggering apoptosis [70,71]. Caspase-9 and Caspase-3 are key members of the cysteine-aspartic acid protease family and are also known as the executors of apoptosis [72,73]. The activation of Caspase-9 can further activate Caspase-3, initiating a series of apoptosis-related reactions that ultimately result in cell apoptosis [74].

Over the past few decades, research on the actions of bioactive components of plants has mainly focused on their cancerpreventing properties. The molecular mechanism of sulforaphane, a bioactive substance in broccoli, for its anti-cancer activity, has been confirmed through several animal and epidemiological studies [75]. Broccoli sprout extract, which contains high amounts of sulforaphane, inhibits carcinoma growth by strongly activating mitochondria-mediated apoptosis and arresting the cell cycle [76]. Additionally, it was confirmed that the combined treatment of NaCl and CaSO4 increased the sulforaphane content in broccoli. In a previous study, it was confirmed that broccoli extract grown with deep sea water minerals exhibited anticancer effects in HT-29 human colon carcinomas [12,77]. 

Substance analysis confirmed an increase in the expression of substances such as 12-oxo-phytodienoic acid, myristic diethanolamine, and aspicilin in NFB. 12-oxo-phytodienoic acid (OPDA), a plant-derived oxylipin, is involved in priming plant responses to stress and, as a biosynthetic precursor of jasmonates (JA), mediates signaling of plant stress responses independently of JA signaling [78]. OPDA induces plant defense responses against insects in rice and is also a substance that regulates stomatal closure in response to drought [79,80]. It is also noteworthy that these OPDAs exhibited particularly cytoprotective effects among all JAs tested in mammalian cells. OPDA treatment was shown to have a cytoprotective effect against HO-induced oxidative stress in SH-SY5Y cells by regulating Nrf2-dependent antioxidant responses [81]. This activation of oxidation-related genes by Nrf2 activation is very similar to sulforaphane and also shows that plant-derived OPDA acts in a similar manner to sulforaphane and can prevent oxidative stress-related diseases [82]. Moreover, studies on the mechanism of action of this oxilipin in plants revealed that OPDA, but not JA, regulates cellular redox homeostasis by binding to cyclophilin20-3 and activating cysteine synthesis in response to cellular stress [83]. According to Carmen González-Bosch’s research, this shows similarities between priming in plants and preconditioning of mammals with phytochemicals and provides information about how sulforaphane and OPDA can modulate stress responses in plants and mammals [82]. Aspicilin, a phenolic compound, was isolated from lichen by Hesse in 1900 [84]. However, the biological significance of aspicilin has not yet been discovered, but it has been identified as a potential inhibitor of Ag85C to inhibit the activity of drug-resistant Mycobacterium tuberculosis [85]. In this way, differences between broccoli samples were confirmed through material analysis, but it appears that further investigation should be conducted into the relationship between the above materials and minerals.

In this study, broccoli extract exhibited a significant inhibitory effect on colon cancer progression and significantly regulated the expression of cell cycle arrest and apoptosis-related factors in NFB. Additionally, the mineral content and content of certain substances tended to increase in NFB. These results suggest that broccoli supplemented with DSWM fertilizer may better control various types of cancer, indicating that consuming mineral-enriched broccoli could potentially delay the progression of colorectal cancer. Furthermore, it demonstrates that DSWM fertilizer may enhance the physiological activity of plants more effectively than trace element fertilizers. This implies that the addition of deep sea water minerals has the potential to increase the physiological vitality of plants, contributing to the observed anticancer effects in broccoli. Furthermore, the benefits of DSWM itself include its potential for environmentally friendly fertilizer production with minimal impact from environmental pollution. This not only applies to broccoli cultivation but also indicates the possibility of enhancing significant effects on the growth and physiological activity of various crops, extending beyond the simple cultivation of broccoli.

However, it may be difficult for the colorectal cancer model used in the experiment to represent all types of colorectal cancer and generalizability to various cancer types may be limited. Additionally, the optimal conditions for the anticancer effect in broccoli cultivation, depending on the specific composition or concentration of deep sea water mineral fertilizer, must also be considered. It is believed that physiological differences or environmental factors in the mice used in the experiment may affect the results. Taking these limitations into account, we plan to focus our follow-up research on supplementing the experimental design and increasing the validity of the results.

## 4. Materials and Methods

### 4.1. Broccoli Preparation

Broccoli was cultivated at the research site of iCOOP Naturaldream Company, located in Yeongin-myeon, Asan-si, Chungcheongbuk-do, Korea, using the Batavia variety. The nutritional management standards were based on the Crop Cultivation Manual provided by the Rural Development Administration of South Korea. To supply the minimum required nutrients for the crops, the input amounts of basal and top dressing and nitrogen (N), phosphorus (P), calcium (Ca), and boron (B) were composed identically. Cultivated broccolis were categorized into four types, depending on the type of nutrient solution fertilizer used. These include Conventional Farming Broccoli (CFB), which uses pure water; Seawater Fertilizer Broccoli (SFB), cultivated using seawater obtained from the front sea area of Asan Bay, South Korea; Trace Element Fertilizer Broccoli (TFB), which incorporates trace element fertilizers; and Naturaldream Fertilizer Broccoli (NFB), cultivated using deep sea water minerals. NFB collects seawater from a depth of 650 m at a point 20 km off the coast of Goseong-gun, Gangwon-do, South Korea, using a microfilter and reverse osmosis, and then moves it to a concentration tank to make concentrated water. Then, the calcium salt and salt content were reduced at a certain rate from 60,000 to 20,000 to 23,000 ppm, and deep sea water minerals (DSWM) were used as fertilizer. NFB manufactured in this way is a fertilizer for crop growth and is registered as an organic farming material by the National Agricultural Products Quality Management Service of the Republic of Korea. All fertilizers were diluted 1000 times and supplied via two foliar applications and five soil drenchings. Mineral components in each fertilizer were analyzed by the Korea Quality Test Institute (Suwon, Gyeonggi-do, Korea), and the analyzed mineral components are shown in Table S1. In addition, in the mineral analysis of the broccoli sample, it underwent pretreatment using the microwave method, a micronutrient test method approved by the Ministry of Food and Drug Safety. The following elements were analyzed using Inductively Coupled Plasma Optical Emission Spectrometry (ICP-OES): sodium, magnesium, zinc, iron, potassium, and calcium. Additionally, the contents of copper and manganese were measured using Inductively Coupled Plasma Mass Spectrometry (ICP-MS) (Table S2). After washing, drying, and freezing at −20 °C, the broccoli was ground into a powder using a freeze dryer. (Tables S1 and S2 are attached in the Supplementary Materials). According to Martins et al., 2022, [86] the daily intake of human broccoli was set at 150 g, and when freeze-dried, 87.3% of moisture was removed, corresponding to 19.05 dry weight. The intake of 19.05 g of broccoli powder (BP) per person (60 kg) corresponds to a dose of 317.5 mg/kg, and when the dose conversion formula is applied between humans and mice, the equivalent animal dose in mice is 3905.25 mg/kg, which is approximately 78 mg BP/mouse [87]. Based on these results, this study also extracted broccoli powder with ethanol and freeze-dried it to produce a daily oral dose of 1.4 g/kg body weight. This dosage was administered for eight weeks (Full text attached in the Supplementary Materials).

### 4.2. Animal Experiment Design

**Figure 12:**
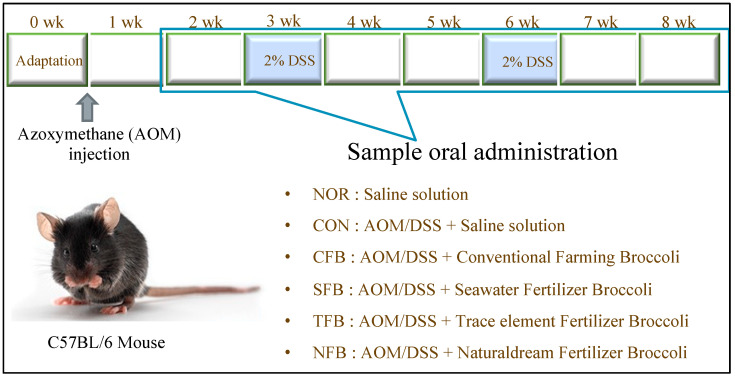
Experimental method used in this study.

The animal experiments were conducted with the agreement of the Animal Experimentation Ethics Committee of CHA University (IACUC220126). Mice used in the experiment were purchased from Orient Bio (Seongnam, Gyeonggi, Korea) at 6-week-old C57BL/6N strain with a body weight of 20 ± 2 g, and maintained under the conditions of 23 ± 2 °C, 55 ± 5% relative humidity for 12 h under a light–dark cycle. After allowing adaptation for a week, each group was classified into six groups of ten mice each, considering body weight: AIN 93G diet group (normal, NOR), AIN 93G diet, and AOM/DSS-induced colorectal cancer group (control, CON), AIN 93G diet with conventional farming broccoli sample and AOM/DSS-induced colorectal cancer group (CFB), AIN 93G diet with seawater fertilizer broccoli sample and AOM/DSS-induced colorectal cancer group (SFB), AIN 93G diet with trace element fertilizer broccoli sample and AOM/DSS-induced colorectal cancer group (TFB), and AIN 93G diet with naturaldream fertilizer broccoli sample and AOM/DSS-induced colorectal cancer group (NFB). AOM (Sigma, St. Louis, MO, USA) was mixed with water, and 10 mg per 1 kg of mouse weight was intraperitoneally administered once to induce colon cancer in C57BL/6N mice. Two weeks after AOM administration, 2% DSS (MPBio, Solon, OH, USA) was administered for the entire week, after which the mice were rested for two weeks. Then, DSS was provided again for a whole week. After nine weeks of this experiment, the mice were fasted for 12 h and sacrificed (Figure 12) [88,89,90].

### 4.3. Assessment of the 2,2-Diphenyl-1-picrylhydrazyl (DPPH) Inhibition Rate in Broccoli

Broccoli samples extracted with methanol were dissolved in DMSO and kept at 4 °C until needed. A 96-well plate was filled with 100 μL of CFB, SFB, TFB, NFB, and methanol and 150 μM of DPPH solution and the reaction was run in the dark for 30 min. To verify the DPPH inhibition rate, the absorbance at 517 nm was measured and computed using the following formula [91]:

$$DPPH\;inhibition\;rate\;(\%)=[1-\frac{\left(SD-SM\right)}{\left(MD-MM\right)}]\times 100$$

SD: Sample + DPPH; SM: Sample + Methanol; MD: Methanol + DPPH; MM: Methanol + Methanol.

### 4.4. Assessment of the Total Phenolic (TP) Content

The Folin–Denis method was used to determine the TP component content. First, 15 µL of Folin–Ciocalteu reagent (Sigma-Aldrich Co., St. Louis, MO, USA) was combined with 5 µL of a specific quantity of broccoli methanol extract and reacted at room temperature for 5 min. The light was then blocked for 40 min at room temperature after adding 40 µL of 7.5% Na_2_CO_3_ solution and 140 µL of distilled water. A Wallac Victor3 1420 Multilabel Counter (Perkin-Elmer, Wellesley, MA, USA) was used to detect absorbance at 765 nm. The standard curve was created using gallic acid as the reference (standard concentration was 0.03125–1 mg/mL), and it was used to quantify the TP content of broccoli [92].

### 4.5. Assessment of the Total Flavonoid (TF) Content

First, 200 μL of diethylene glycol was added to 20 μL of sample extract and set aside for 5 min at room temperature. Then, 20 μL of 1N NaOH was dispensed with vortexing, and the block was heated for 1 h at 37 °C. The absorbance at 420 nm was measured using a Wallac Victor3 1420 Multilabel Counter. The flavonoid content was determined using the standard calibration curve generated by drawing the standard curve using Quercetin (Sigma-Aldrich Co., St. Louis, MO, USA) as the reference (standard concentration is 0–1280 μg/mL).

### 4.6. Sample Collection

The body weight of the mice was measured and recorded every Wednesday at a fixed time. After eight weeks of oral administration, the mice were euthanized using CO_2_ gas, and blood was collected from the heart, followed by weighing of the liver, spleen, testes, kidneys, and colon. To determine whether colon cancer was induced, the colon’s length and the number of tumors were checked. Blood and spleen were stored on ice for subsequent experiments, and the colon and liver were transferred into liquid nitrogen and stored in a refrigerator at −80 °C. 

### 4.7. Histopathological Studies

Histopathological studies were conducted on the colon and spleen tissues. After thoroughly washing the colon and spleen tissue removed from the mouse with PBS, the tissue sample was sliced into 2–3 mm thick pieces and fixed in 10% formalin solution for ≥24 h. Then, the specimens were embedded in paraffin and sliced into 4-μm thick sections before being stained with H&E. The sections were examined by an experienced observer under a Nikon Microscope ECLIPSE50i (Nikon Inc., Tokyo, Japan) equipped with an infinity camera (SPOT RT741 Slider Color, Diagnostic Instruments, Sterling Heights, MI, USA).

### 4.8. Measurement of Inflammatory Cytokines Using Enzyme-Linked Immunosorbent Assay (ELISA)

After the blood was centrifuged at 3000 rpm for 15 min, the serum was collected and stored at −80 °C for later use. Splenocytes were isolated using a 40 μm cell strainer and treated with red blood cell lysis buffer. The suspension was centrifuged at 1000 rpm for 3 min, and the pellet containing the dissociated cells was resuspended in RPMI 1640 medium (Welgene Inc., Daegu, Republic of Korea) containing 1% penicillin-streptomycin solution (Gibco BRL., Rockville, MD, USA) and 10% inactivated fetal bovine serum (Welgene Inc., Daegu, Republic of Korea). The cells were cultured for 24 h, and the supernatant was used in the experiments. Blood serum and splenocyte supernatants were then tested for IL-1β, IL-6, TNF, IFN-γ, IL-12, and IL-10 concentrations using ELISA kits (ELISA kits, BioLegend, San Diego, CA, USA). The detailed experimental technique was conducted according to the manufacturer’s instructions [12].

### 4.9. Quantitative Reverse Transcription Polymerase Chain Reaction (qRT-PCR)

After colon and liver tissues from the experimental animals were washed with saline, RNA was isolated from them using Trizol (Invitrogen, Carlsbad, CA, USA) and dissolved in 0.1% diethyl pyrocarbonate (DEPC). Total dissolved RNA was quantified using Nano Drop ND-1000 (Nano Drop Technologies Inc., Wilmington, DE, USA), and cDNA was synthesized using Superscript II reverse transcriptase (Invitrogen, Carlsbad, CA, USA). The synthesized cDNA was analyzed for gene expression using a Bio-Rad CFX-96 real-time thermal cycler system (Bio-Rad, Hercules, CA, USA). Table 2 shows the gene types and primer sequences used [12].

### 4.10. Western Blotting

Colon and liver tissues of experimental animals were homogenized using a 1 mL Radio-immunoprecipitation assay (RIPA, Invitrogen, Carlsbad, CA, USA) buffer, and proteins were separated via centrifugation at 13,000 rpm for 5 min at 4 °C. The separated proteins were quantified using the Bradford assay. The extracted proteins were separated via sodium dodecyl sulfate–polyacrylamide gel electrophoresis (SDS-PAGE) and transferred into a polyvinylidene fluoride (PVDF, Bio-Rad, Hercules, CA, USA) membrane. Nonspecific proteins were blocked with 5% skimmed milk containing phosphate-buffered saline with Tween 20 (PBST). After blocking, the PVDF membrane was washed thrice with PBST and once with PBS, and the primary antibody was reacted overnight at 4 °C. Then, the PVDF membrane was washed thrice with PBST and once with PBS, and the secondary antibody was reacted for 2 h at room temperature. For Bax, Bcl-2, caspase-3, caspase-9, p53, IL-6, NF-κB p65, IκB-α, TNF, and β-actin, Santa Cruz (Dallas, TX, USA) primary antibodies were used, and the proteins were detected using an Amersham Imager 680 (GE Healthcare Life Sciences, Chicago, IL, USA). The experiment was conducted at least three times.

### 4.11. Measurement of Natural Killer Cell Activity

Isolated spleen cells from experimental animals were cultured in a medium for 24 h. The medium used was Roswell Park Memorial Institute (RPMI) 1640 medium (Welgene Inc., Daegu, Republic of Korea), which was supplemented with 10% heat-inactivated fetal bovine serum (FBS, GIBCO, Grand Island, NY, USA) and 1% penicillin–streptomycin (PS) solution (Welgene, Gyeongsan-si, Gyeongsangbuk-do, Republic of Korea). Subsequently, the spleen cells were used as effector cells, whereas YAC-1 cells (NK-sensitive cell line) were used as target cells. The ratio of effector cells to target cells was adjusted to 5:1. Simultaneously, broccoli extract samples were added at 2 mg/mL concentration. Then, incubation was continued for 4 h, and NK cell activity was evaluated by measuring lactate dehydrogenase (LDH) produced by the cells at 450 nm using an EZ-LDH cytotoxicity assay kit (Dogenbio, Seoul, Korea) to determine cytotoxicity [12].

### 4.12. Metabolomic Analysis and Multivariate Statistical Analysis

Broccoli metabolites extracted with 70% methanol were analyzed using ultra-performance liquid chromatography–quadrupole-time of flight (UPLC-Q-TOF) mass spectrometry (MS). The extracts were injected into an Acquity UPLC BEH C18 column (2.1 mm × 100 mm, 1.7 μm; Waters, Milford, MA, USA). The mobile phase consisted of water with 0.1% formic acid (FA) (A) and ACN with 0.1% FA (B) at a flow rate of 0.35 mL/min for 12 min. The eluted metabolites were ionized by positive or negative electrospray ionization mode and detected using Q-TOF MS with 3 kV (positive mode) or 2.5 kV (negative mode) of capillary voltages, 40 V of sampling cone voltage, 900 L/h (positive mode) or 800 L/h (negative mode) of desolvation flow rate, 400 °C of desolvation temperature, and 100 °C of the source. MS/MS spectra were obtained using collision energy ramps of 10 to 20 eV. MS data were collected from m/z 50 to 1500, the peak width at 5% height with an intensity threshold of 10,000, peak-to-peak baseline noise of 1, and noise elimination set to 6 using MarkerLynx version 4.1 software (Waters, Milford, MA, USA). The peaks were aligned and mass spectra were normalized. Metabolites were identified using the UNIFI version 1.9.2.045 software (Waters) connected to various online databases. Multivariate statistical analysis with SIMCA-P+ version 12.0.1 (Umetrics, Umeå, Sweden) was used to analyze the processed MS data sets using partial least squares discriminant analysis (PLS-DA).

### 4.13. Statistical Analysis

Graph Pad Prism 9.4.1 (GraphPad, San Diego, CA, USA) was used to analyze the data, and the experimental results are presented as mean ± standard deviation. Duncan’s multiple range test was used to verify the significance between each group using one-way ANOVA. *p* < 0.05 was considered statistically significant, and all experimental data were analyzed using the SPSS v18 statistical software package (SPSS Inc., Westlands, Hong Kong).

## 5. Conclusions

In this study, broccoli grown using deep sea water minerals as fertilizer had higher antioxidant effects and mineral content than seawater and mineral fertilizers, and a significantly increasing trend was confirmed through material analysis. In addition, the colon and spleen tissue in AOM/DSS-treated mice were restored to a level similar to that in normal mice, and it also significantly regulated the expression of inflammatory cytokines in the spleen and serum and appeared to suppress the expression of inflammatory factors in liver tissue. Finally, it had a significant recovery effect on a mouse model of colon cancer by alleviating colon cancer and inducing intestinal cell cycle arrest and apoptosis. Overall, broccoli grown using deep sea water minerals as fertilizer showed higher anti-inflammatory and anticancer effects than broccoli grown using other cultivation methods, making it a potential crop for the prevention and treatment of colon cancer. Additionally, this is not simply limited to broccoli, but the use of deep-ocean water mineral fertilizers supposedly can increase crop functionality in other crop cultivation.

## Figures and Tables

**Table 1:** Weight of each organ in mice (g).

	NOR	CON	CFB	SFB	TFB	NFB
Liver	0.96 ± 0.06 ^b^	1.07 ± 0.08 ^a^	1.00 ± 0.10 ^ab^	0.94 ± 0.08 ^b^	0.94 ± 0.08 ^b^	0.92 ± 0.05 ^b^
Spleen	0.05 ± 0.01 ^a^	0.18 ± 0.04 ^b^	0.16 ± 0.11 ^b^	0.08 ± 0.01 ^a^	0.07 ± 0.02 ^a^	0.06 ± 0.01 ^a^
Kidney	0.31 ± 0.02 ^a^	0.26 ± 0.02 ^c^	0.27 ± 0.02 ^bc^	0.28 ± 0.01 ^bc^	0.28 ± 0.01 ^abc^	0.30 ± 0.02 ^ab^
Testis	0.18 ± 0.02 ^a^	0.14 ± 0.02 ^b^	0.14 ± 0.02 ^b^	0.16 ± 0.02 ^b^	0.16 ± 0.01 ^b^	0.19 ± 0.01 ^a^

NOR: saline solution; CON: AOM/DSS + saline solution; CFB: AOM/DSS + Conventional Farming Broccoli 1.4 g/kg body weight (b.w.); SFB: AOM/DSS + Seawater Fertilizer Broccoli 1.4 g/kg b.w.; TFB: AOM/DSS + Trace element Fertilizer Broccoli 1.4 g/kg b.w.; NFB: AOM/DSS + Naturaldream Fertilizer Broccoli 1.4 g/kg b.w. Means with different letters (a–c) on the same raw are significantly different (*p* < 0.05) using Duncan’s multiple range test.

**Table 2:** Primer sequences for the RT-qPCR assay.

Gene Name	Primer Sequence
*NF-* *κ* *B p50*	F: 5′-CACCTAGCTGCCAAAGAAGG-3′
	R: 5′-GCAGGCTATTGCTCATCACA-3′
*NF-* *κ* *B p65*	F: 5′-ATGGCAGACGATGATCCCTAC-3′
	R: 5′-CGGAATCGAAATCCCCTCTGTT-3′
*IFN-* *γ*	F: 5′-GCTTTGCAGCTCTTCCTCAT-3′
	R: 5′-GTCACCATCCTTTTGCCAGT-3′
*COX-2*	F: 5′-GGTGCCTGGTCTGATGATG-3′
	R: 5′-TGCTGGTTTGGAATAGTTGCT-3′
*iNOS*	F: 5′-ATGGCTTGCCCCTGGAA-3′
	R: 5′-TATTGTTGGGCTGAGAA-3′
*IL-6*	F: 5′-ATGAAGTTCCTCTCTGCAA-3′
	R: 5′-AGTGGTATCCTCTGTGAAG-3′
*IL-12*	F: 5′-CATCGATGAGCTGATGCAGT-3′
	R: 5′-CAGATAGCCCATCACCCTGT-3′
*IL-10*	F: 5′-CCAAGCCTTATCGGAAATGA-3′
	R: 5′-TTTTCACAGGGGAGAAATCG-3′
*IL-4*	F: 5′-TCAACCCCCAGCTAGTTGTC-3′
	R: 5′-TGTTCTTCGTTGCTGTGAGG-3′
*p53*	F: 5′-ATGGAGGAGCCGCAGTCAGA-3′
	R: 5′-TGCAGGGGCCGCCGGTGTAG-3′
*p21*	F: 5′-ATGTCAGAACCGGCTGGGG-3′
	R: 5′-GCCGGGGCCCCGTGGGA-3′
*Bim*	F: 5′-AGATCCCCGCTTTTCATCTT-3′
	R: 5′-TCTTGGGCGATCCATATCTC-3′
*Bad*	F: 5′-CAATGACCCCTTCATTGACC-3′
	R: 5′-GACAAGCTTCCCGTTCTCAG-3′
*Bak*	F: 5′-TCTGGCCCTACACGTCTACC-3′
	R: 5′-AGTGATGCAGCATGAAGTCG-3′
*Bax*	F: 5′-TGCTTCAGGGTTTCATCCAG-3′
	R: 5′-GGCGGCAATCATCCTCTG-3′
*Bcl-2*	F: 5′-AAGATTGATGGGATCGTTGC-3′
	R: 5′-GCGGAACACTTGATTCTGGT-3′
*Caspase-9*	F: 5′-CTAGTTTGCCCACACCCAGT-3′
	R: 5′-CTGCTCAAAGATGTCGTCCA-3′
*Caspase-3*	F: 5′-TTTTTCAGAGGGGATCGTTG-3′
	R: 5′-CGGCCTCCACTGGTATTTTA-3′
*GAPDH*	F: 5′-AGGTCGGTGTGAACGGATTTG-3′
	R: 5′-GGGGTCGTTGATGGCAACA-3′

## Data Availability

Data are contained within the article.

## Supplementary Materials

The supporting information can be downloaded at: https://www.mdpi.com/article/10.3390/ijms25031650/s1.
