# Supplementary material for: Broccoli Cultivated with Deep Sea Water Mineral Fertilizer Enhances Anti-Cancer and Anti-Inflammatory Effects of AOM/DSS-Induced Colorectal Cancer in C57BL/6N Mice
Source: Int J Mol Sci. 2024 Jan 29;25(3):1650. doi: 10.3390/ijms25031650 (PMC10855752; doi:10.3390/ijms25031650)
Supplement: Supplementary file 1 [file ijms-25-01650-s001.zip › Supplementary file_prepare broccoli samples for oral administration_materials and methods_broccoli preparation.pdf]

Supplementary data on how to prepare broccoli samples for oral administration

According to Martins et al., 2022, [26] the daily intake of human broccoli was set at 150 g, and when freeze-dried, 87.3% of moisture was removed, corresponding to 19.05 dry weight. The intake of 19.05 g of Broccoli Powder (BP) per person (60 kg) corresponds to a dose of 317.5 mg/kg, and when the dose conversion formula is applied between humans and mice, the equivalent animal dose in mice is 3,905.25 mg/kg, which is approximately 78 mg BP/mouse [27]. Additionally, there was no problem even at a dose ten times higher through a pilot experiment, and assuming that adults consume thrice weekly, 780-mg BP thrice weekly corresponds to an average daily intake of 334.23mg BP/mouse. When the average daily food intake of the mice was 5 g, broccoli corresponded to 6.7% of the daily feed intake. In this experiment, when broccoli was freeze-dried, 87.84% of the water was removed, and the broccoli weight was reduced to 12.16% compared with the original weight. When this was calculated in the same way as above, it was calculated as 373.92 mg/mL, and 70% ethanol was added ten times to prepare it as an oral administration sample, followed by stirring twice for 24 h. When the extracted broccoli was powdered using a vacuum concentrator, the yield was 37.03%. Upon calculating the daily oral dosage, it was determined as 1.4-g/kg body weight. This dosage was administered for eight weeks.

References

26. Martins, T.; Oliveira, P.A.; Pires, M.J.; Neuparth, M.J.; Lanzarin, G.; Felix, L.; Venancio, C.; Pinto, M.L.; Ferreira, J.; Gaivao, I.; et al. Effect of a Sub-Chronic Oral Exposure of Broccoli (*Brassica oleracea* L. Var. *Italica*) By-Products Flour on the Physiological Parameters of FVB/N Mice: A Pilot Study. *Foods* **2022**, *11*, doi:10.3390/foods11010120.

27. Nair, A.B.; Jacob, S. A simple practice guide for dose conversion between animals and human. *J Basic Clin Pharm* **2016**, *7*, 27-31, doi:10.4103/0976-0105.177703.
