# Supplementary material for: Broccoli Cultivated with Deep Sea Water Mineral Fertilizer Enhances Anti-Cancer and Anti-Inflammatory Effects of AOM/DSS-Induced Colorectal Cancer in C57BL/6N Mice
Source: Int J Mol Sci. 2024 Jan 29;25(3):1650. doi: 10.3390/ijms25031650 (PMC10855752; doi:10.3390/ijms25031650)
Supplement: Supplementary file 1 [file ijms-25-01650-s001.zip › Supplementary Table S1.pdf]

Table S1. Mineral content analysis results for each fertilizer.

mg/kg	Na	Mg	Ca	K	Fe	P(PO_4)	Si	Cl	N	B
**Seawater Fertilizer**	6879	808	282	261	1.11	-	-	1700	-	-
**Trace element Fertilizer**	1339	3432	698	9784	23938	7.37	8182	-	-	-
**Naturaldream Fertilizer**	9565	60000	49.5	11588	N.D	10.8	0.61	14700	27	179
