# Supplementary material for: Broccoli Cultivated with Deep Sea Water Mineral Fertilizer Enhances Anti-Cancer and Anti-Inflammatory Effects of AOM/DSS-Induced Colorectal Cancer in C57BL/6N Mice
Source: Int J Mol Sci. 2024 Jan 29;25(3):1650. doi: 10.3390/ijms25031650 (PMC10855752; doi:10.3390/ijms25031650)
Supplement: Supplementary file 1 [file ijms-25-01650-s001.zip › Supplementary Table S2.pdf]

Table S2. Mineral contents of broccoli. CFB: Conventional Farming Broccoli; SFB: Seawater Fertilizer Broccoli; TFB: Trace element Fertilizer Broccoli; NFB: Naturaldream Fertilizer Broccoli. Means with the different letters (a-d) above the bars are significantly different ($p < 0.05$) by Duncan's multiple range test.

mg/kg	Na	Mg	Ca	K	Zn	Fe	Mn	Cu
**CFB**	1040.81 ± 15.47[c]	2056.50 ± 16.37[b]	3884.87 ± 17.65[a]	31219.95 ± 164.75[b]	52.95 ± 0.36[b]	58.63 ± 0.23[c]	29.78 ± 0.94[b]	4.41 ± 0.09[b]
**SFB**	1242.42 ± 1.88[a]	1954.40 ± 8.08[c]	3939.72 ± 7.93[a]	31177.77 ± 77.16[b]	48.02 ± 0.33[d]	60.15 ± 0.15[c]	28.90 ± 1.25[b]	4.14 ± 0.11[c]
**TFB**	829.95 ± 4.00[d]	1772.48 ± 12.10[d]	3629.82 ± 2.00[b]	31203.18 ± 153.23[b]	54.60 ± 0.38[a]	64.43 ± 1.44[b]	32.90 ± 1.03[a]	4.19 ± 0.08[c]
**NFB**	1180.15 ± 9.86[b]	2193.48 ± 20.73[a]	3933.98 ± 59.71[a]	32415.70 ± 190.01[a]	50.47 ± 0.14[c]	68.00 ± 1.35[a]	27.91 ± 0.99[b]	4.76 ± 0.13[a]
